# Enhancing the Solubility of Co-Formulated Hydrophobic Drugs by Incorporating Functionalized Nano-Structured Poly Lactic-*co*-glycolic Acid (*nf*PLGA) During Co-Precipitation

**DOI:** 10.3390/pharmaceutics17010077

**Published:** 2025-01-08

**Authors:** Mohammad Saiful Islam, Somenath Mitra

**Affiliations:** Department of Chemistry and Environmental Science, New Jersey Institute of Technology, Newark, NJ 07102, USA; mi238@njit.edu

**Keywords:** co-formulation, functional *nf*PLGA, incorporation, antisolvent technology, co-precipitation, dissolution, aqueous solubility

## Abstract

**Background/Objectives**: The co-formulation of active pharmaceutical ingredients (APIs) is a growing strategy in biopharmaceutical development, particularly when it comes to improving solubility and bioavailability. This study explores a co-precipitation method to prepare co-formulated crystals of griseofulvin (GF) and dexamethasone (DXM), utilizing nanostructured, functionalized polylactic glycolic acid (*nf*PLGA) as a solubility enhancer. **Methods**: An antisolvent precipitation technique was employed to incorporate *nf*PLGA at a 3% concentration into the co-formulated GF and DXM, referred to as DXM-GF-*nf*PLGA. The dissolution performance of this formulation was compared to that of the pure drugs and the co-precipitated DXM-GF without *nf*PLGA. **Results**: Several characterization techniques, including electron microscopy (SEM), RAMAN, FTIR, TGA, and XRD, were used to analyze the *nf*PLGA incorporation and the co-precipitated co-formulations. The inclusion of *nf*PLGA significantly enhanced the dissolution and initial dissolution rate of both GF and DXM in the DXM-GF-*nf*PLGA formulation, achieving a maximum dissolution of 100%, which was not attained by the pure drugs or the DXM-GF formulation. The incorporation of *nf*PLGA also reduced the amount of time taken to reach 50% (T_50_) and 80% (T_80_) dissolution. T_50_ values decreased from 52 and 82 min (for pure DXM and GF) to 23 min for DXM-GF-*nf*PLGA, and the T_80_ improved to 50 min for DXM-GF-*nf*PLGA, significantly outpacing the pure compounds. Furthermore, incorporating *nf*PLGA into the crystal structures greatly accelerated the dissolution rates, with initial rates reaching 650.92 µg/min for DXM-GF-*nf*PLGA compared to 540.60 µg/min for DXM-GF, while pure GF and DXM showed lower rates. **Conclusions**: This work demonstrates that *nf*PLGA incorporation enhances dissolution performance by forming water channels within the API crystal via hydrogen-bonding interactions. This innovative *nf*PLGA incorporation method holds promise for developing hydrophobic co-formulations with faster solubility and dissolution rates.

## 1. Introduction

Solubility plays a critical parameter in drug delivery [1,2], especially for drugs classified under Biopharmaceutical Classification System (BCS) Class II and IV [3,4] due to their poor solubility and low bioavailability [5,6]. These compounds constitute a substantial proportion—approximately 40 to 70 percent [7]—of new drug entities (NCE)/candidates in development stages [8,9,10]. Consequently, significant hurdles may be encountered while designing and delivering such drug products, particularly when it comes to formulating strategies to overcome solubility or permeability challenges [10,11]. Low solubility often results in unstable therapeutic concentrations, necessitating the implementation of developmental strategies [12] to enhance drug solubility and permeability [1,13,14]. Consequently, optimizing drug solubility can significantly contribute to improved therapeutic outcomes [15].

Significant efforts have been dedicated to overcoming the solubility challenges of hydrophobic drugs [16]. Conventional techniques for solubility enhancement include particle size reduction [17] through methods like micronization [18] and nanoparticle formation [10,19,20], solid dispersion techniques [21], co-solvents [22], complexation [23], salt formation [24], pH adjustment [25], surfactant use [26], hydrotropic [27], lipid-based formulations [28], nano-emulsions [29], spray drying [30], and solvent selection [17,31]. Formulations with solubilizing agents such as polymers and surfactants have also been used for dissolution enhancement [32]. Additionally, various materials like nanostructured dendrimers, micelles, carbon nanotubes, graphene, graphene oxide, quantum dots [33,34], proteins [35], viruses [36], ceramics [37,38], metals, semiconductors [39], lipids [28], polymeric thin films [40], hydrogels [41], and amorphous or crystalline surfaces are utilized in different types of drug formulations to enhance their solubility [42,43].

Co-formulation presents an attractive strategy that combines multiple drugs into a single oral solid or injectable product [44,45]. For poorly soluble drug components, co-formulation has been shown to enhance bioavailability [44,46]. Additionally, it is useful for developing combination therapies [47,48]. Recent advancements in this field include approved fixed-dose combinations like Pertuzumab, Trastuzumab, and Hyaluronidase for HER-2-positive breast cancer [49], stable formulations combining durvalumab (Imfinzi^®^, anti-PD-L1) and tremelimumab (anti-CTLA-4) [50], as well as co-formulated stable solid dispersions of Artemether (ARTM) and Lumefantrine (LUMF) using optimized drug–polymer–surfactant blends via hot-melt extrusion [51], and spherical cocrystallization via direct compression to improve solubility and bioavailability [52]. Co-formulated drugs can streamline treatment protocols, optimize drug delivery, and reduce manufacturing costs [44]. Moreover, co-formulated drug formulations hold clinical potential for treating complex diseases. Previous research has shown that multiple drugs can work synergistically within the GI-to-blood circulation system, minimizing adverse effects [53]. This supports the idea that co-formulation strategies are crucial for modern pharmaceutical development [54].

Co-formulation via co-precipitation can promote intermolecular interactions at the molecule’s surfaces, leading to faster solubility and improved bioavailability profiles [55]. Thus, co-precipitation can enhance the physicochemical properties of combined hydrophobic drugs [56,57]. Co-formulation that involves the antisolvent precipitation technique is promising for drug-delivery research [58]. Our previous study has explored incorporating graphene oxide (nGO) [59], carbon nanotubes [60], functionalized nanostructured polylactic acid (PLA) [61], and polylactic glycolic acid (PLGA) [62] into hydrophobic APIs to enhance dissolution. Although nGOs have hydrophilic functional groups on their surfaces, facilitating interaction with water to enhance dissolution [63,64], they are not FDA-approved and may pose toxicity risks. Alternatively, we have investigated the use of functionalized nanoparticles of PLGA [65], referred to as *nf*PLGA. They are not water-soluble by themselves, but contain hydrophilic functionalized groups on their surfaces [66,67], such as carboxyl and/or hydroxyl, which promote the aqueous solubility of the insoluble drug crystals through hydrophilic interactions. This incorporation helps to facilitate faster dissolution.

The objective of this research is to incorporate *nf*PLGA to co-precipitate the hydrophobic API co-formulations to fabricate drug–drug-*nf*PLGA composites with enhanced dissolution properties. Antifungal Griseofulvin, which is a BCS II highly hydrophobic drug with a solubility of 0.00864 mg/L [68], and dexamethasone (DXM), which is a BCS-IV classified corticosteroidal and anti-inflammatory, 9-fluoro-glucocorticoid drug that is practically insoluble in water with a solubility profile of 0.080 mg/mL [69,70], are used to form co-formulated composites with enhanced solubility. The combination of an antifungal drug and a steroid is commonly used to treat infections accompanied by inflammation, making this combination highly significant from a therapeutic standpoint.

## 2. Materials and Methods

### 2.1. Materials

Griseofulvin and dexamethasone were purchased from Sigma Aldrich (St. Louis, MO, USA). Poly lactic-*co*-glycolic acid polymer was purchased from Polysciences Inc. (Warrington, PA, USA). Acetone was bought from Sigma Aldrich, and sulfuric acid and nitric acid were bought from Fisher Scientific supplier (Thermo Fisher Scientific Inc., Waltham, MA, USA). The source of 1-Octanol was also Sigma Aldrich (St. Louis, MO, USA), and the purified Milli-Q water was collected from NJIT York centers Milli-Q plus system.

### 2.2. Fabrication of nfPLGA

Synthesis of *nf*PLGA particles was carried out following a previously published method [61,62]. A multimode microwave-accelerated reaction system, specifically the CEM Microwave Reactor (MARS-5, Matthews, NC, USA), was used for the acid oxidation of PLGA. Ground PLGA polymer was mixed with a 1 M concentration of sulfuric acid (H_2_SO_4_) and nitric acid (HNO_3_) solution in a 3:1 ratio. This mixture comprised 200 mg of PLGA and 60 mL of the acid solution.

The acid-dispersed PLGA mixture was transferred to a microwave sample holder, which was then tightly closed and sealed to ensure an airtight environment. The microwave reactor was operated under specific conditions: a standard control type program, power set to 800 W at 80% intensity, temperature maintained at 60 °C, and pressure at 200 psi. The reaction was allowed to run for 60 min, with a hold time of 10 min.

After completing the 60 min microwave-accelerated reaction, the acid-treated and microwave-irradiated PLGA samples were vacuum-filtered through a 0.2-micron PTFE membrane filter, washed with milli-Q water, and vacuum-dried for 48 h. Once dried, the functionalized PLGA powder/crystalline particles were dispersed in milli-Q water. This dispersion was then subjected to sonication using a probe sonicator. The samples were sonicated in 60 min intervals while maintaining a controlled temperature to ensure that the sample temperature remained at room temperature. The resulting sonicated particles are referred to as *nf*PLGA.

### 2.3. Preparation of Co-Formulated DXM-GF-nfPLGA

An antisolvent precipitation method was employed to precipitate co-formulated drugs, specifically DXM-GF and DXM-GF-*nf*PLGA composites, following a previously outlined process [59,60]. Acetone was used to dissolve the 200 mg of DXM and 200 mg of GF containing DXM-GF mixture (1:1), while *nf*PLGA (12 mg), a nano-functionalized polymer, was dissolved in acetone to produce a clear polymer solution. The DXM-GF mixture in acetone was subjected to bath sonication, and the polymer solution was gradually added dropwise into the drug mixture. This process of adding the polymer and continuing sonication for up to 10 min ensured proper mixing of the components. Afterward, the combined drug solution was removed from the sonication bath and left at room temperature for 30 min to stabilize. Subsequently, the co-formulation was placed in an ice bath, and milli-Q water was added dropwise to induce the antisolvent effect. The milli-Q water acted as the antisolvent, and after sufficient addition, a milky suspension of the DXM-GF-*nf*PLGA co-formulation was formed, which later resulted in the precipitation of a bulk amount of the product. The precipitated particles were then filtered using a 0.2-micron PTFE membrane filter and vacuum-dried in an oven at room temperature for 48 h to achieve finely dried particles. Additionally, the co-formulation without *nf*PLGA (DXM-GF) was prepared separately to distinguish the effects of *nf*PLGA incorporation.

### 2.4. Characterization of Co-Formulated DXM-GF-nfPLGA

Various analytical characterization techniques were employed to investigate and characterize the formulated work. The hydrodynamic Z-average size and zeta potential of the functionalized and nano-sized *nf*PLGA particles were measured using a Dynamic Light Scattering (DLS) system (Malvern Nano ZS, Model: ZEN 3600, Worcestershire, UK). A JEOL JSM-7900F scanning electron microscope (SEM) (JEOL, Tokyo, Japan) was utilized to image the drug crystal formulations, and the samples were carbon-coated using an EMS Quorum coater. Optimized operating conditions were applied during SEM imaging, including a 10 mm working distance and an accelerating voltage of 5 kV. Aztec software (AZtecLive 5.1) was used to produce the elemental identification and elemental mapping of the co-formulation.

A PerkinElmer 8000 Model TGA instrument (Shelton, CT, USA) was used to analyze the decomposition profile of the co-formulated drugs and quantify *nf*PLGA incorporation. For the TGA, approximately 10 mg of powder samples were placed in a ceramic sample holder and heated in a furnace under a nitrogen flow rate of 20 mL/min. The heating rate was set to 10 °C/min, and the temperature ranged from 30 to 700 °C. The melting point and purity of the co-formulated drugs were determined using a Differential Scanning Calorimeter (PerkinElmer DSC 6000, Shelton, CT, USA). In the DSC analysis, the operating temperature was between 30 and 300 °C at a 10 °C/min heating rate, and the sample amount was between 5 and 10 mg. Raman spectral intensity was measured with a ThermoFisher Scientific DXR2xi Raman imaging microscope instrument (Madison, WI, USA), employing a 532 nm laser wavelength and full-frequency acquisition mode 3800–200 cm^−1^ region. Powder X-ray diffraction (PXRD) was performed to confirm the crystalline identity of the co-formulated drugs using the PANalytical EMPYREAN XRD (Malvern, UK) instrument with a Cu Kα radiation source. The diffraction intensity was recorded over a 2θ range of 5–70°. A HighScore Plus (version 5.2) software was used for analysis. Fourier-transform infrared (FTIR) analysis was performed (using diamond ATR) with an Agilent Cary 670 Benchtop spectrometer (Santa Clara, CA, USA) to assess the functional properties of the drugs and their co-formulation. The analysis was conducted with ResolutionsPro (version 5.4.0.3389) software, utilizing 64 scans per sample and a resolution of 4 cm^−1^.

The aqueous solubility of the drug samples was determined as follows. The drug formulation (50 mg) in 100 mL milli-Q water in a glass vial was stirred in water for 24 h using a magnetic stirrer to reach equilibrium [71,72]. Afterward, the mixture was filtered, and the resulting solution was analyzed via UV absorption. The octanol–water partition coefficient was measured as follows. For this experiment, 50 mg of the drug sample was added to a 1:1 mixture (50 mL each) of water (aqueous phase) and octanol (organic phase) for octanol–water partitioning. The mixture was stirred for one hour to allow for partitioning between the organic and aqueous phases and to achieve equilibrium. Afterward, ultracentrifugation separated the two phases, and the aqueous phase was collected. The drug concentration in the aqueous phase was then determined using a UV-Vis spectrometer, and the concentration in the octanol phase was determined by subtracting the aqueous phase value. Then, the logP was calculated based on these concentrations [73,74].

The in vitro dissolution test for the co-formulated drugs was conducted using the USP apparatus II paddle system with sink condition, in accordance with the United States Pharmacopeia (USP) dissolution method 〈711〉 [75,76,77]. The dissolution performance was assessed using the Symphony 7100 Distek instrument (North Brunswick, NJ, USA) following the established protocol. Simulated gastric fluid (SGF), prepared to mimic stomach conditions at pH 1.4, was used as the dissolution medium. This was achieved by mixing 900 mL of 0.1 N HCl to obtain the desired pH, which was then added to the dissolution bath. The experiment was performed under optimized conditions, including a temperature of 37 ± 0.5 °C and a paddle rotation speed of 75 rpm, over a 4 h duration. Samples were collected at predetermined intervals. Drug particles (100 mg) were initially dispersed in a small volume of water and introduced into the dissolution bath using a syringe, allowing them to circulate freely in the medium. Aliquots of approximately 2 mL were withdrawn at time points of 5, 10, 20, 30, 50, 80, 120, 150, 180, and 240 min. The collected samples were filtered through 0.2-micron sterile PTFE syringe filters and transferred into cuvettes for UV-Vis analysis. Drug concentrations were quantified using an Agilent 8453 UV-Vis spectrophotometer (Santa Clara, CA, USA), with calibration performed at 240 nm and 295 nm.

## 3. Results and Discussion

### 3.1. Characteristics of DXM-GF-nfPLGA

**Figure 1:**
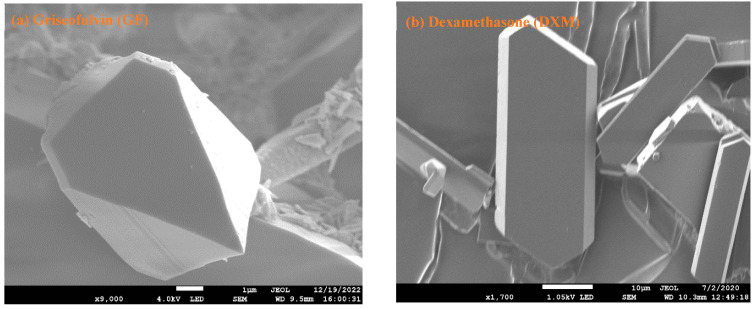

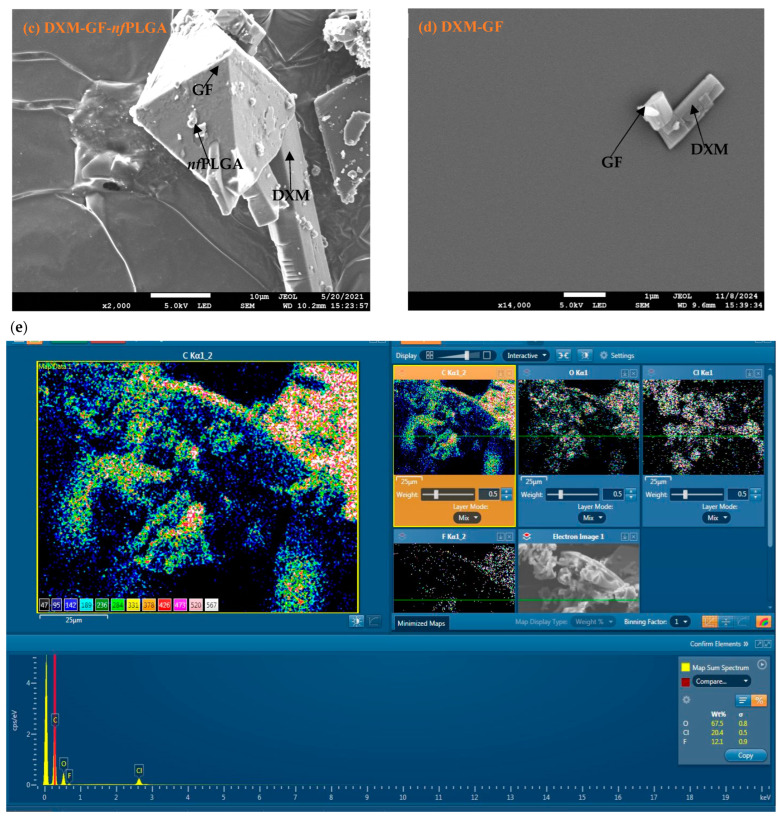
SEM images of (**a**) Pure GF, (**b**) pure DXM, (**c**) DXM-GF-*nf*PLGA co-formulation (carbon coated), (**d**) DXM-GF, and (**e**) EDS elemental mapping for co-formulation of DXM-GF-*nf*PLGA.

The SEM images of pure GF, DXM, DXM-GF, and DXM-GF-*nf*PLGA composites are presented in Figure 1a–d. It is evident from Figure 1c that the crystal structure of the single drug GF and DXM remains intact within the physical integration, and the *nf*PLGA attaches to the surface of the co-formulated drugs. Additionally, the *nf*PLGA particles are expected to provide hydrophilic linkages over the drug surfaces, which may produce a water channel to the co-formulated drugs that will lead to high dispersibility and aqueous solubility. The analyses suggest that the co-formulated drugs can retain their structural and morphological integrity upon *nf*PLGA incorporation during the antisolvent process.

The elemental point ID and mapping (EDS) analysis, illustrated in Figure 1e, confirms the distribution of constituent elemental concentrations within the co-formulations derived from individual drugs [78,79]. The analysis reveals the presence of oxygen at 67.5 wt% (from both GF and DXM), chlorine at 20.4 wt% (from GF), and fluorine at 12.1 wt% (from DXM). The figure displays a color legend with distinct color codes corresponding to numerical values, indicating the concentration gradient of specific elements or a combination of elements from lowest to highest. Furthermore, the EDS mapping highlights two distinct crystals that appear to be stacked or attached.

Table 1 presents the water solubility and octanol–water partition coefficient (log P) of the drugs and their co-formulated counterparts. The solubility of the co-formulated drugs with incorporated *nf*PLGA was 0.064 mg/mL, surpassing that of DXM-GF. Furthermore, the log P for the co-formulated drugs was 1.15, indicating a moderately low value due to the enhanced hydrophilicity provided by *nf*PLGA incorporation. The data in Table 1 also reveal that DXM-GF-*nf*PLGA demonstrated an increased zeta potential of −30.2 mV, reflecting improved stability in the aqueous medium. This enhanced stability is attributed to the surface charge of *nf*PLGA particles in the dispersion. Conversely, the pure drugs or DXM-GF alone exhibited a lower zeta potential of −19.7 mV, underscoring their limited ability to achieve similar stability.

**Figure 2:**
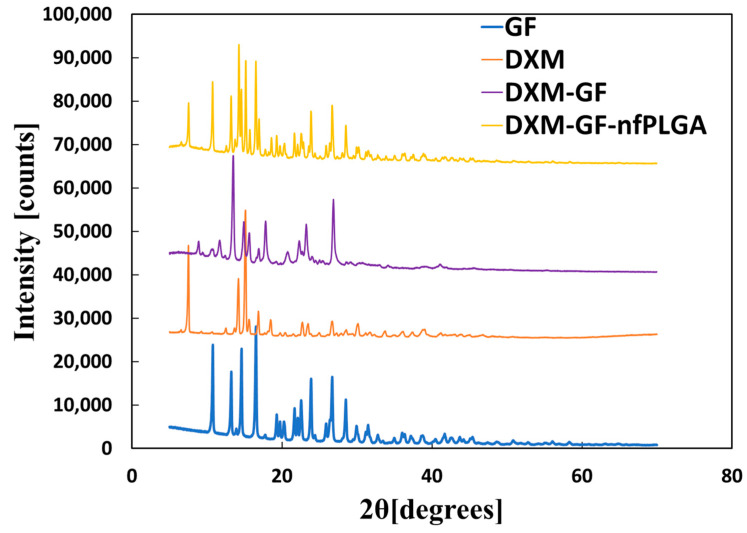
X-ray diffraction (XRD) analysis data for co-formulated drugs’ formulation.

**Figure 3:**
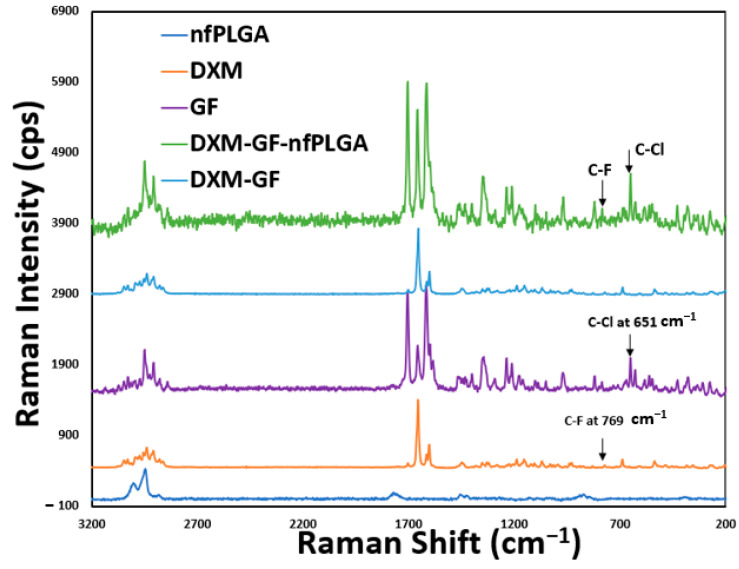
RAMAN analysis data for co-formulated drugs’ formulation.

**Figure 4:**
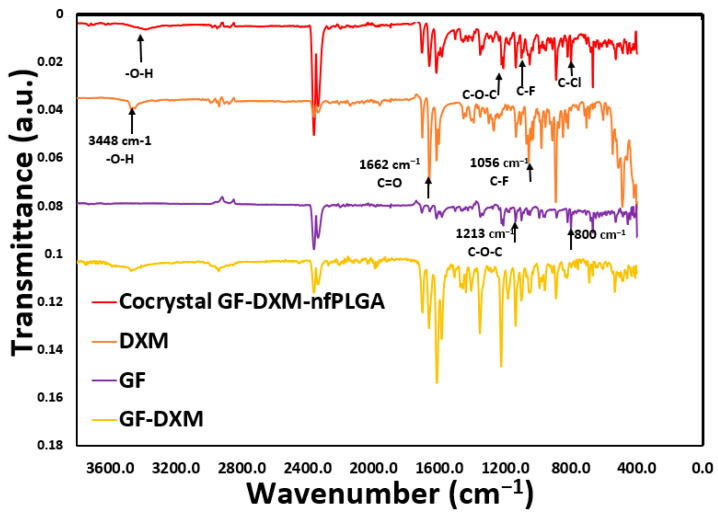
FTIR analysis of co-formulation of drug formulations.

An important consideration was whether the DXM or GF structure was altered during the co-formulation process. Figure 2, Figure 3 and Figure 4 display the XRD, Raman, and FTIR spectral intensity analysis of pure DXM, GF, DXM-GF, and DXM-GF-*nf*PLGA co-formulations. Raman spectra showed distinct peaks for dexamethasone at the C-F stretch (769 cm^−1^) and for griseofulvin at the C–Cl stretch (651 cm^−1^), both of which were present in the co-formulation, indicating that their polymorphism remained unchanged. Additionally, X-ray diffraction (XRD) of the co-formulated drugs revealed intensity peaks at different 2θ angles (Supplementary Files contain detailed XRD analysis) corresponding to those of DXM and GF, further supporting the idea that their crystallinity was not altered. In the Fourier-transform infrared spectroscopy (FTIR) analysis, characteristic absorption bands for GF, such as C–O–C (1213 cm^−1^) and C–Cl (800 cm^−1^), as well as for DXM, including –O–H (3448 cm^−1^), C=O (1662 cm^−1^), and C–F (1056 cm^−1^), were detected in the co-formulated drugs. Importantly, no changes were observed in the characteristic functional group intensities of the individual drugs after co-formulation. FTIR analysis thus confirmed the presence of both DXM and GF in the co-formulated products, with no significant structural alterations.

**Figure 5:**
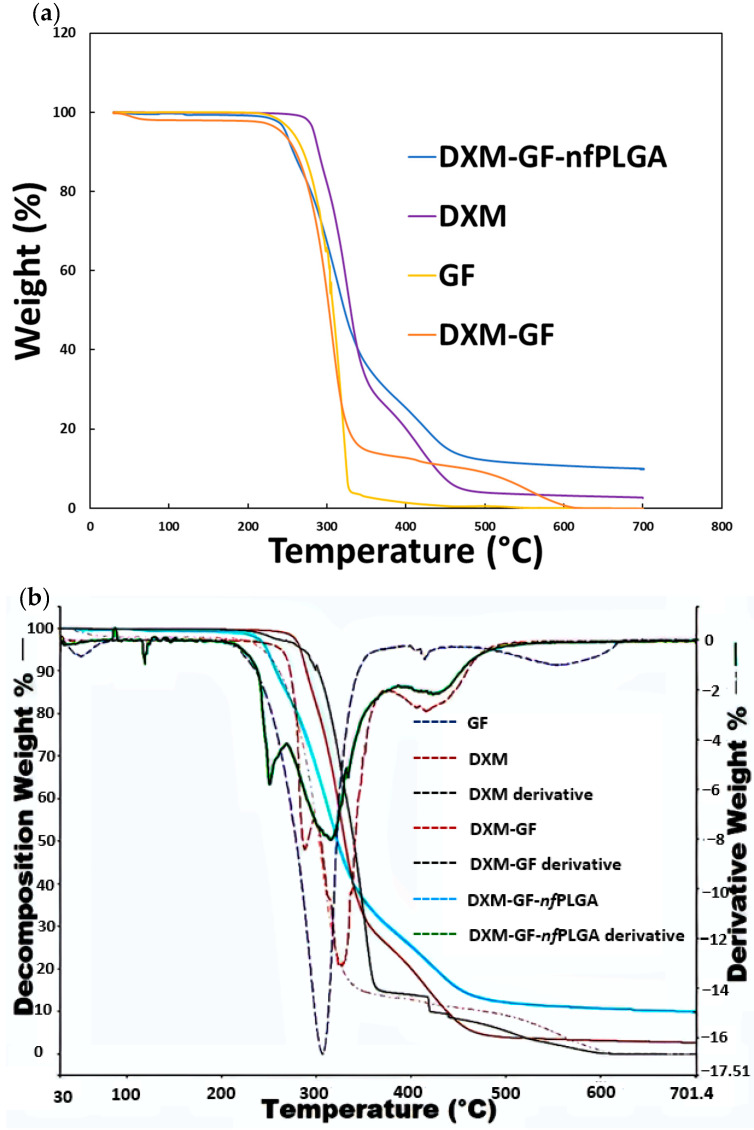
(**a**) TGA of the co-formulated drugs to determine the percentage of *nf*PLGA incorporation and (**b**) antisolvent crystal GF and DXM % from first-derivative curve analysis.

**Figure 6:**
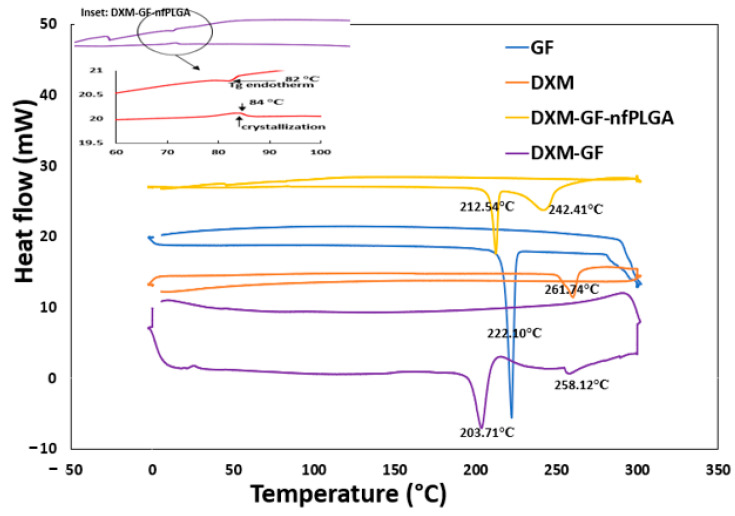
DSC analysis of pure compounds and co-formulations.

In Figure 5a, thermogravimetric analysis (TGA) was used to study the *nf*PLGA incorporation into the co-formulated drugs. The analysis shows that the major decomposition temperature for the co-formulation occurred between 250 and 400 °C. The final formulation contained 66% GF, 31% DXM, and 3% *nf*PLGA. The DXM-GF without *nf*PLGA incorporation contained 60% GF and 40% DXM. Figure 6 shows the differential scanning calorimetry (DSC) for the co-formulated drugs’ endothermic peak and the melting point measurement. The DSC thermograms showed a change in the heat capacity and the glass transition at approximately 82 °C and 84 °C [80], followed by a slightly lower endothermic crystallization peak shift between 242.24 °C (DXM) and ~212.5 °C (GF), respectively, from the original drug [81]. The presence of *nf*PLGA and co-precipitation of the two drugs appear to alter the melting points (Table 1) slightly.

### 3.2. In Vitro Drug Dissolution Analysis

The in vitro drug dissolution and release tests were performed in accordance with the USP-42 dissolution protocol. In this procedure, 0.1 N HCl with a pH of 1.4 was used as the dissolution medium to simulate gastric conditions. The improved dissolution observed in the *nf*PLGA-incorporated drugs and/or co-formulated drug formulations is hypothesized to result from the hydrophilic surface properties of *nf*PLGA, which facilitate interactions with drug molecules, leading to the formation of inter- and intramolecular hydrogen bonds.

**Figure 7:**
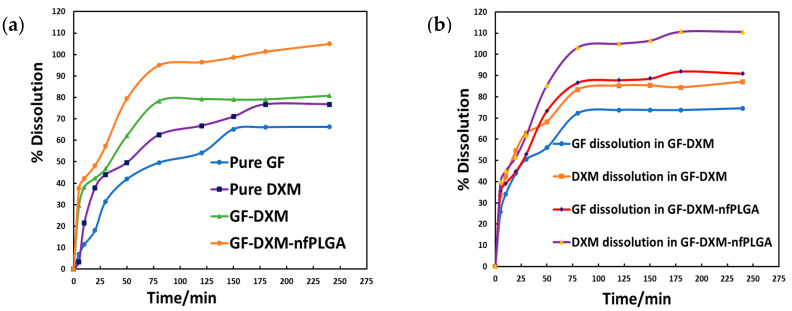
(**a**) Dissolution profile of co-formulated drugs and (**b**) % of individual drug dissolution profile into the co-formulated drugs.

Figure 7a is the dissolution profile for DXM, GF, DXM-GF, and DXM-GF-*nf*PLGA. It is evident that *nf*PLGA incorporation led to an enhanced dissolution rate and aqueous solubility, which was attributed to intermolecular interaction with water. The maximum dissolution for GF and DXM reached 100% in DXM-GF-*nf*PLGA, which was not achievable for the pure DXM, GF, or DXM-GF. DXM-GF showed some enhancement over the pure drugs but was still significantly lower than DXM-GF-*nf*PLGA.

Table 2 presents key dissolution property data, including the improved initial dissolution rate and the time required to achieve 50% dissolution (T_50_) and 80% dissolution (T_80_), respectively. Pure DXM and GF had poor water solubility; the T_50_ was 52 and 82 min, respectively. DXM-GF and DXM-GF-*nf*PLGA showed enhanced dissolution compared to the pure APIs; the overall T_50_ in DXM-GF was 34 min, whereas GF and DXM had T_50_s of 29 and 19 min, respectively. With DXM-GF-*nf*PLGA, the overall T_50_ reduced to 23 min, while those of GF and DXM were 27 and 18 min, respectively. The enhancement was most marked for T_80_, where pure DXM and GF never reached 80% dissolution, and in DXM-GF, only DXM could achieve an 80% dissolution mark. However, DXM-GF-*nf*PLGA showed an excellent overall T_80_ of 50 min, where GF was at 61 min and DXM at 44 min. The initial rate of dissolution was also an important consideration. Pure GF and DXM had initial dissolution rates of 110.27 µg/min and 180.90 µg/min, respectively. The co-formulated DXM-GF showed an initial dissolution rate of 540.60 µg/min, with GF and DXM at 220.61 µg/min and 290.74 µg/min, respectively. However, the initial dissolution rate was significantly enhanced by the incorporation of *nf*PLGA and reached as high as 650.92 µg/min, with 266.81 µg/min for GF and 325.74 µg/min for DXM.

## 4. Conclusions

This research presents a novel approach to pharmaceutical formulation, leveraging nano-functionalized PLGA (*nf*PLGA) to enhance the dissolution and potentially improve the bioavailability of hydrophobic co-formulated drugs. By combining antisolvent precipitation technology with *nf*PLGA hydrophilic functionalization achieved through microwave-induced oxidation, this study offers an innovative solution to the persistent challenge of poor drug solubility. The findings demonstrate that the DXM-GF-*nf*PLGA co-formulation achieved complete dissolution (100%) for both GF and DXM, exhibiting superior dissolution kinetics compared to formulations without *nf*PLGA. The incorporation of *nf*PLGA not only reduced the time spent in the gastric environment but also significantly shortened the amount of time taken to reach 50% and 80% dissolution (T_50_ and T_80_) while improving the initial dissolution rate, addressing a critical obstacle in drug delivery for poorly soluble drugs.

A comprehensive suite of characterization techniques, including XRD, Raman spectroscopy, FTIR, SEM, and in vitro dissolution tests, was employed to assess the structural, morphological, and dissolution properties of the DXM-GF-*nf*PLGA formulation. The results reveal that the inclusion of *nf*PLGA and the multi-drug antisolvent precipitation method markedly enhance solubility and dissolution, likely improving bioavailability. Accelerated initial dissolution rates further confirm that *nf*PLGA plays a pivotal role in enhancing aqueous solubility through hydrogen-bonding interactions, facilitating the formation of water channels. This work validates the hypothesis that *nf*PLGA is an effective agent for promoting faster solubility and dissolution of hydrophobic drugs in co-formulations, offering significant potential for pharmaceutical applications and addressing a major limitation in drug development.

## Figures and Tables

**Table 1:** Physicochemical properties of the formulated drugs’ co-formulation.

Formulations	Aqueous Solubility (µg/mL)	Zeta Potential (mV)	logP	Melting Point (°C)
Pure DXM	89.00	−17.2	1.96	261.74
Pure GF	8.64	−15.4	2.16	222.10
DXM-GF	83.70	−19.7	1.75	258.12 (DXM) 203.71 (GF)
DXM-GF-*nf*PLGA	128.70	−30.2	1.15	242.41 (DXM) 212.54 (GF)

**Table 2:** The dissolution profile of the co-formulated drugs.

Formulations	50% Dissolution Time (T_50_)	80% Dissolution Time (T_80_)	Initial Dissolution Rate [0 to 20 min] (µg/min)	Maximum Dissolution (%)
Pure GF	82	Undissolved	110.27	66.2
Pure DXM	52	Undissolved	180.90	76.8
DXM-GF	34	100	540.60	81.0
GF in DXM-GF	29	Undissolved	220.61	74.55
DXM in GF-DXM	19	72	290.74	87.05
DXM-GF-*nf*PLGA	23	50	650.92	105
GF in DXM-GF-*nf*PLGA	27	61	266.81	90.9
DXM in DXM-GF-*nf*PLGA	18	44	325.74	110.6

## Data Availability

The original contributions presented in this study are included in the article/Supplementary Material. Further inquiries can be directed to the corresponding author.

## Supplementary Materials

The following supporting information can be downloaded at: https://www.mdpi.com/article/10.3390/pharmaceutics17010077/s1, Coformulation XRD analysis.
