# Supplementary material for: Enhancing the Solubility of Co-Formulated Hydrophobic Drugs by Incorporating Functionalized Nano-Structured Poly Lactic-co-glycolic Acid (nfPLGA) During Co-Precipitation
Source: Pharmaceutics. 2025 Jan 8;17(1):77. doi: 10.3390/pharmaceutics17010077 (PMC11768099; doi:10.3390/pharmaceutics17010077)
Supplement: Supplementary file 1 [file pharmaceutics-17-00077-s001.zip › pharmaceutics-3344295-supplementary.pdf]

Supplementary data_Coformulation_XRD Analysis

a) ***nf*****PLGA**

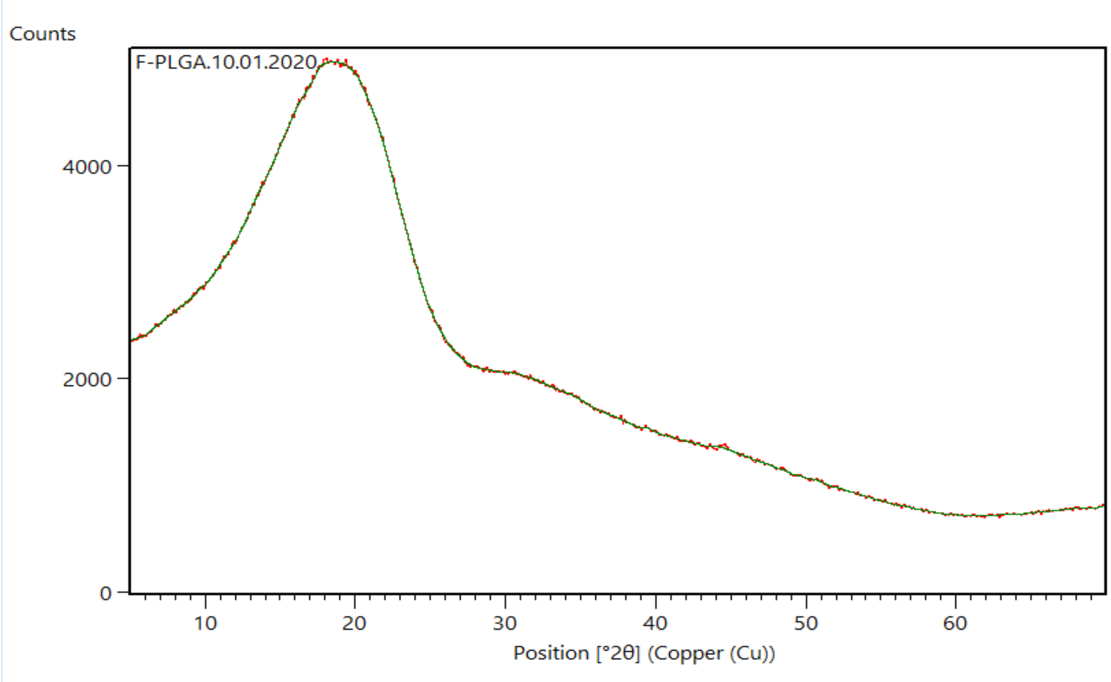

b) GF

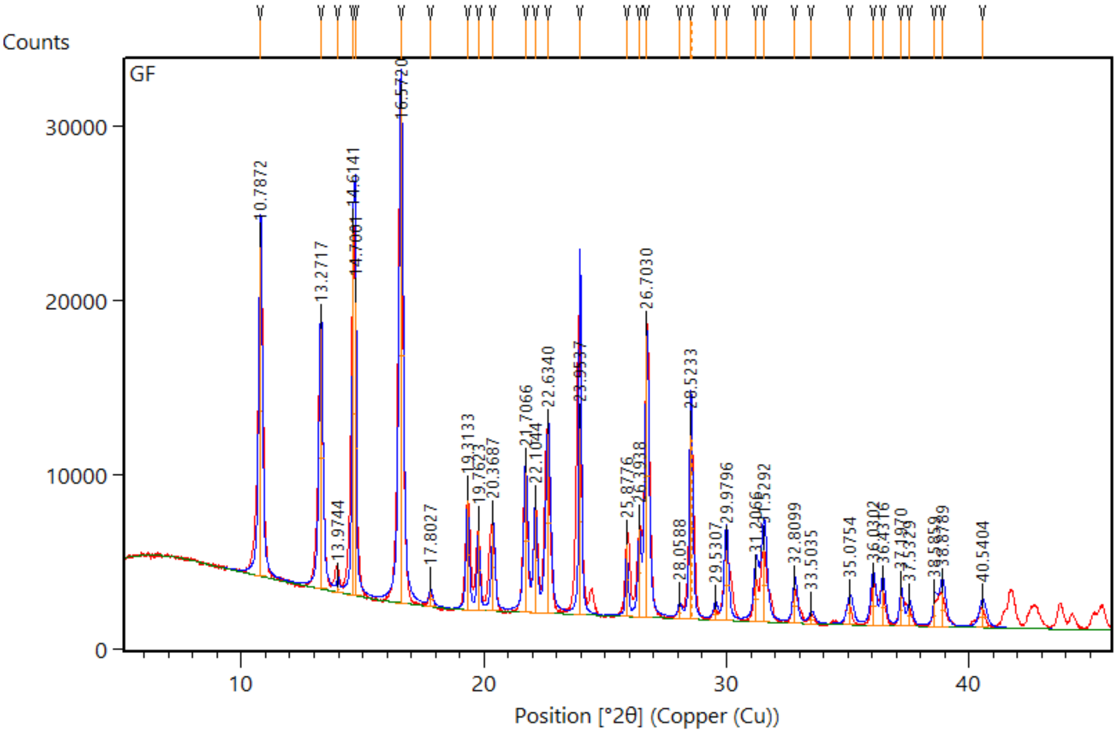

c) DXM

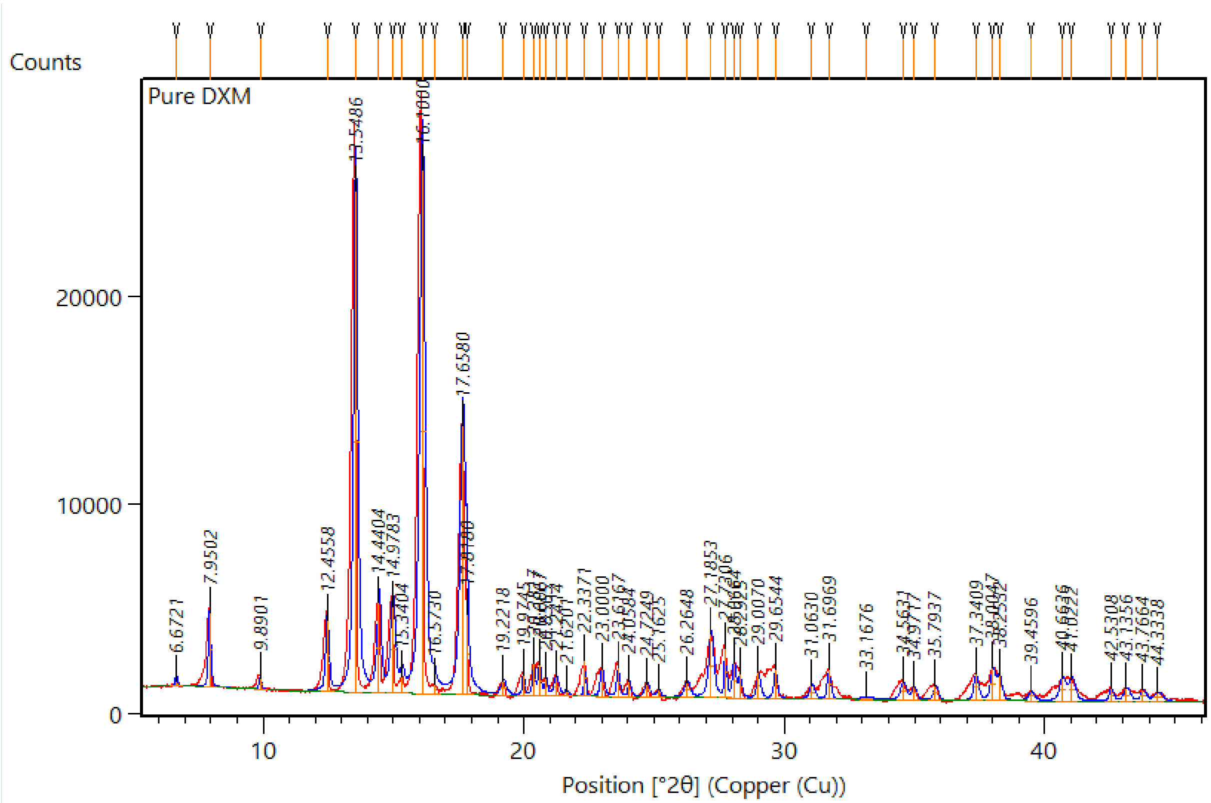

Counts
Pure DXM
20000
10000
0
10
20
30
40
Position [°2θ] (Copper (Cu))
6.6721
7.9502
9.8901
12.4558
13.5486
14.4404
14.9783
15.3404
16.1000
16.5730
17.6580
19.2218
22.3371
23.0000
24.7249
25.1625
26.2648
27.1853
29.0070
29.6544
31.0630
31.6969
33.1676
35.7937
37.3409
38.2532
39.4596
41.0222
42.5308
43.1356
43.7664
44.3338

d) GF-DXM

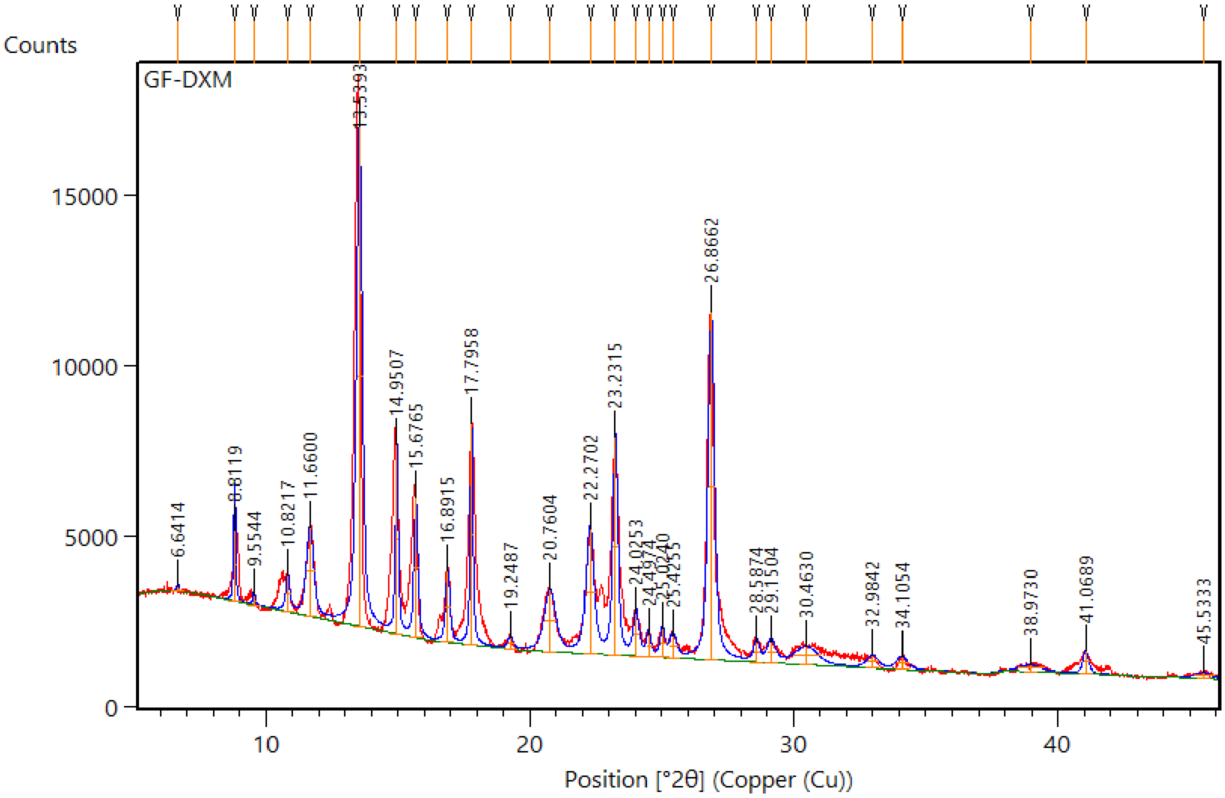

Counts
GF-DXM
15000
10000
5000
0
10
20
30
40
Position [°2θ] (Copper (Cu))
6.6414
9.5544
10.8217
11.6600
13.5393
14.9507
15.6765
16.8915
17.7958
19.2487
20.7604
22.2702
23.2315
24.0253
26.8662
28.5874
29.1504
30.4630
32.9842
34.1054
38.9730
41.0689
45.5333

e) GF-DXM-*nf*PLGA

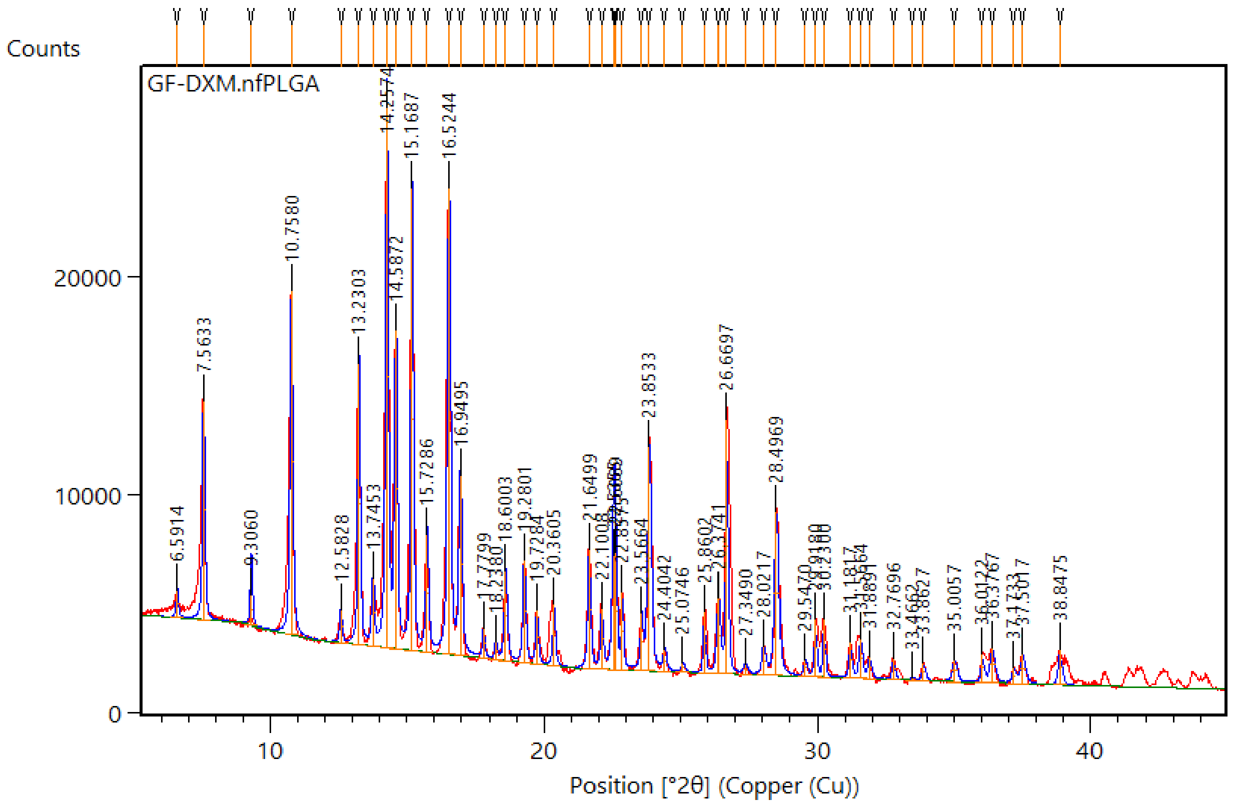

Discussion about the above XRD analysis in figure a-e:

The differences in GF and DXM crystal structure can/may affect the position of XRD peaks during coformulation. In GF, the peak at lower angles (10°–20°) indicates a characteristic crystalline structure. In DXM, the sharp peaks at around 10°–25° indicate the well-defined crystalline nature of dexamethasone. In DXM-GF, slight peak shifts in 10°–25° compared to DXM may be due to some interaction between molecular or physical adsorption between DXM and GF. Meanwhile, in DXM-GF-*nf*PLGA, the 10°–25° peaks are comparatively sharp, and slight peak shifts are indicated due to the incorporation of *nf*PLGA into DXM-GF coformulations.
